# A common variant of RIP3 promoter region is associated with poor prognosis in heart failure patients by influencing SOX17 binding

**DOI:** 10.1111/jcmm.14408

**Published:** 2019-05-31

**Authors:** Dong Hu, Jin Huang, Senlin Hu, Ying Zhang, Shiyang Li, Yang Sun, Chenze Li, Guanglin Cui, Dao Wen Wang

**Affiliations:** ^1^ Division of Cardiology, Department of Internal Medicine Tongji Hospital, Tongji Medical College, Huazhong University of Science and Technology Wuhan China; ^2^ Hubei Key Laboratory of Genetics and Molecular Mechanisms of Cardiological Disorders Wuhan China; ^3^ Division of Cardiology Affiliated Hospital of Guizhou Medical University Guiyang China

**Keywords:** genetics, heart failure, prognosis, receptor‐interacting protein kinase 3

## Abstract

Receptor‐interacting protein kinase 3 (RIP3) is a key determinant of necroptosis and participates in ischaemia—and oxidative stress‐induced necroptosis, myocardial remodelling and heart failure (HF). In this study, we tested the hypothesis that common variants in RIP3 gene were associated with the risk and prognosis of HF in the Chinese Han population. By re‐sequencing and luciferase assays, we identified a common functional variant in the RIP3 promoter region. The rs3212247‐T allele suppressed RIP3 promoter activity by facilitating transcription factor SOX17 binding, but not the C allele. We further recruited 2961 control participants and 3194 HF patients who underwent a mean follow‐up of 19 months (6‐31 months) for this study. Rs3212247 and another missense variant rs3212254 were genotyped. Although rs3212247 did not significantly associate with increased risk of HF (odds ratio = 1.00, 95% CI = 0.92‐1.08, *P* = 0.91), it raised the risk for cardiovascular death and cardiac transplantation (hazard ratio = 1.47, 95% CI = 1.13‐1.91, *P* = 0.004). Moreover, participants carrying the rs3212247 CC genotype had higher plasma levels of RIP3 than those carrying the TT or TC genotype (p for trend = 0.02) in New York Heart Association class III HF group. No association was found between the RIP3 missense variant rs3212254 and risk or prognosis of HF after adjustment for traditional risk factors. In conclusion, genetic variant in RIP3 promoter region is associated with increased RIP3 transcription, thus contributed to the poor prognosis of HF patients. Clinical Trial Registration: https://www.clinicaltrials.gov/ct2/show/NCT03461107?term=03461107&cond=Heart+Failure&cntry=CN&rank=1. Unique identifier: NCT03461107.

## 1 INTRODUCTION

Chronic heart failure (HF) is a complex clinical syndrome caused by various factors including coronary heart disease, hypertension, valvular heart disease and idiopathic dilated cardiomyopathy.^1^ Although great advances have been achieved in medical and surgical therapy recent decades, the 5‐ and 10‐year mortality rates of HF remain high.^2^, ^3^ As the ageing of population and HF risk factors increase, HF has become a serious challenge for the public health.^3^, ^4^ Better understanding of the genetic basis of HF may shed light on the prevention, diagnosis and treatment of HF.^5^, ^6^

Recently, genome wide association studies have successfully identified multiple genes associated with the risk of HF.^7^, ^8^, ^9^, ^10^ However, only a few studies focused on the association of genetic variation with its prognosis.^4^, ^11^ Up to now, genetic loci and related genes associated with mortality of HF have remained rarely discovered and need more investigation. Thus, we speculated that there may exist other functional variants which could modify the prognosis of HF.

Necroptosis is a form of necrosis strictly regulated by distinct signalling pathway.^12^ Substantial evidence has demonstrated that RIPK1‐RIPK3‐MLKL axis represented the core components for tumour necrosis factor‐induced necroptosis both in human and mouse, which could be abolished by Receptor‐interacting protein kinase 3 (RIP3)‐depletion.^12^, ^13^, ^14^ Many pathological processes including malignant melanoma,^15^ intestinal tumourigenesis^16^ and Abdominal Aortic Aneurysms^17^ also have the participation of RIP3. Importantly, RIP3 showed strong expression in hearts of murine and human, which implied the importance of this protein for the function of myocardium.^18^, ^19^, ^20^ Recently, studies have shown that necroptosis played a vital role in ischaemia—and oxidative stress—induced myocardial remodelling and HF, which could be alleviated by RIP3‐depletion.^18^, ^21^ Based on these findings, we hypothesized that RIP3 gene variation may affect its expression and modify the susceptibility and prognosis of HF.

In the present study, we re‐sequenced the regulatory region of RIP3 gene including the promoter and 5'UTR region of 200 control participants to identify putative functional variants. The underlying mechanism was explored. Ultimately, a total of 3194 HF patients and 2961 control participants were genotyped to investigate association between the variants and the occurrence and prognosis of HF.

Here, we demonstrated that rs3212247‐C allele in the promoter of RIP3 gene destroyed the binding of sox17, which could repress the expression of ripk3. Moreover, participants carrying the rs3212247 CC genotype had higher plasma levels of RIP3 than those carrying the TT or TC genotype (p for trend = 0.02) in New York Heart Association (NYHA) class III HF group and the plasma RIP3 levels of NYHA III and IV groups were significantly higher compared with NYHA II and control groups, which suggest positive correlation between ripk3 level and severity of HF. Importantly, population of HF carrying rs3212247‐CC genotype showed poorer prognosis assessed by cardiovascular death combined with cardiac transplantation (hazard ratio = 1.47, 95% CI = 1.13‐1.91, *P* = 0.004).

## 2 MATERIALS AND METHODS

### 2.1 Study population

This study was approved by Ethics Committee of Tongji Hospital and the written informed consents were obtained from all participants. The investigation conformed to the principles of the Declaration of Helsinki. A total of 3394 HF patients were recruited between January 2009 and August 2017 in Cardiology Division of Tongji Hospital in Wuhan. At the last analysis, 200 patients failed to be followed up and the study follow‐up compliance rate was 94.1% (3194/3394). The primary end points were defined as cardiovascular deaths or cardiac transplantation. Details on diagnostic and exclusion criteria of HF, data collection and definition of risk factors are provided online. Ethnically and geographically matched 2961 individuals without evidence of HF were included as controls. Anthropometric measurements, clinical characteristics and clinical events were recorded at planned follow‐up clinic visits, from questionnaires, medical records and telephone calls. The clinical characteristics of the populations are summarized in Table 1.

**Table 1:** Baseline characteristics of the study population

Characteristics	Sequencing healthy population	HF Study	
		Control population	HF population
	Controls (n = 200)	Controls (n = 2961)	Cases (n = 3194)
Men (%)	50	45	65
Age (y)	59.05 ± 9.37	58.6 ± 10.2	56.0 ± 13.5*
Glucose (mmol/L)	5.08 ± 0.46	5.14 ± 0.44	6.78 ± 3.22*
TC (mmol/L)	4.41 ± 0.88	4.93 ± 0.96	3.91 ± 1.15*
TG (mmol/L)	1.44 ± 0.90	1.45 ± 1.00	1.44 ± 1.16
HDL (mmol/L)	1.30 ± 0.33	1.46 ± 0.35	0.99 ± 0.45*
LDL (mmol/L)	2.44 ± 0.70	2.77 ± 0.79	2.38 ± 0.87*
Hypertension (%)	0	58	77
Diabetes (%)	0	0	30
Hyperlipidaemia (%)	0	35	18
Smoking status (%)	0	0	36
β‐blocker use (%)	0	0	53

Note

Data are expressed as means ± SD or percentages.

Abbreviations: HDL, high‐density lipoprotein; LDL, low‐density lipoprotein; TC, total cholesterol; TG, triglyceride.

* *P* < 0.05 cases vs controls.

### 2.2 Genetic variation screening

**Figure 1:**
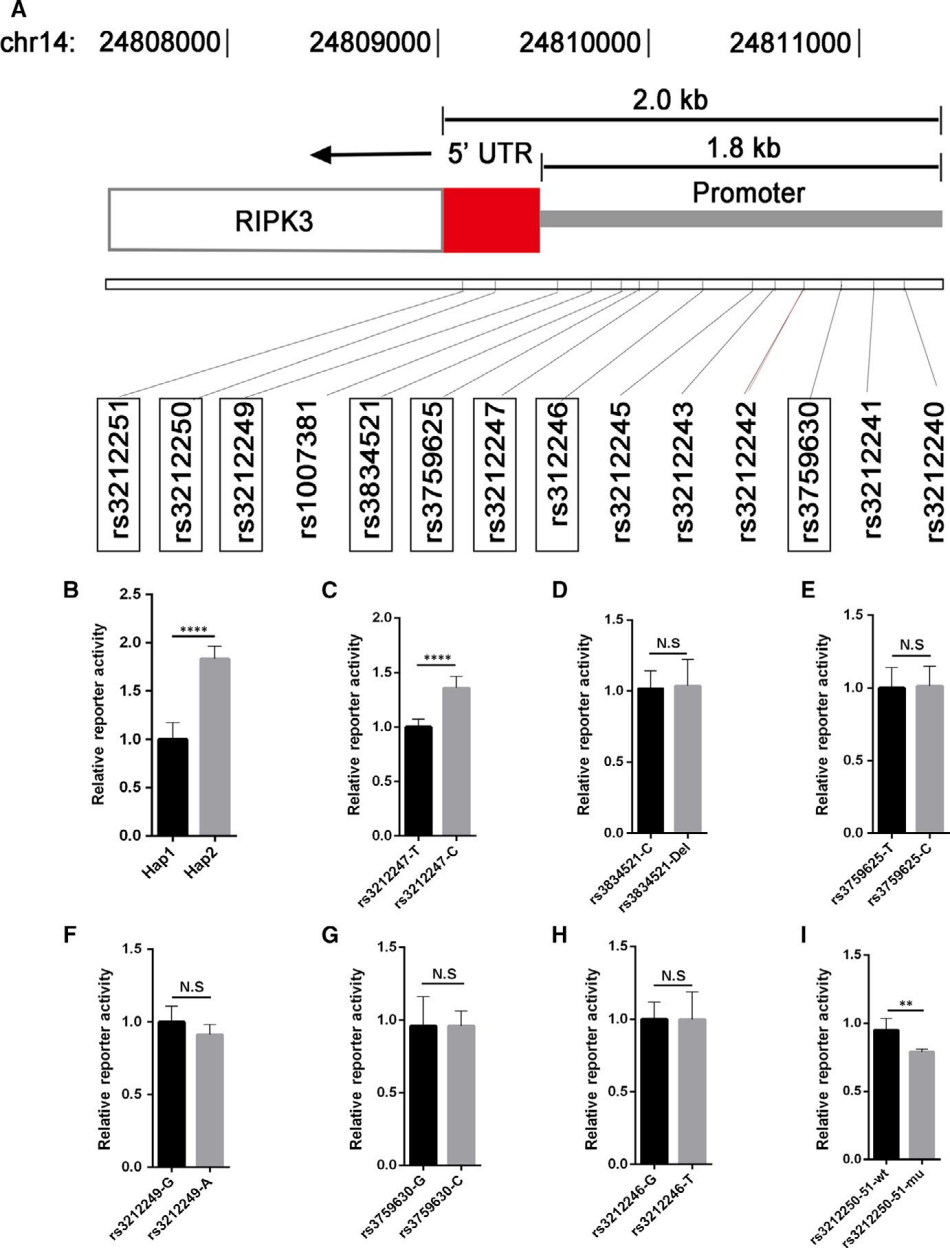
rs3212247 is responsible for haplotype‐specific difference in transcriptional activity. (A), Map of single‐nucleotide polymorphisms (SNPs) in the promoter and 5'UTR of Receptor‐interacting protein kinase 3 genotyped in 200 healthy individuals. The eight SNPs (indicated with boxes), which were predicted to be possible functional at RegulomeDB, comprise a single haplotype and define the 1.8 and 0.2 kb in promoter and 5'UTR, respectively. (B), Firefly luciferase expression from constructs transfected into AC16. Both the major (Hap1) and minor (Hap2) haplotypes of the 1.8 kb in promoter region were subcloned into PGL3‐basic vector. (C‐I), Luciferase assays for rs3212247, rs3834521, rs3759625, rs3212249, rs3759630, rs3212246 rs3212250‐51 (rs3212250 and rs3212251 were combined as a haplotype as they are close enough) were conducted using AC16. Approximately 250‐bp regions encompassing corresponding variants were cloned into PGL3‐basic vector. Luciferase activity was measured 48 h after transfection and was normalized against Renilla luciferase activity. Values are mean ± SE of three independent experiments each corresponding to at least six replicates. **P < 0.01, ****P < 0.0001, NS, not significant

Genomic DNA was extracted from peripheral venous blood samples of 200 control participants. Common variants in the promoter and 5'UTR region of RIP3 were identified by Sanger sequencing. Polymerase chain reaction products were amplified from regions 1.8 kb upstream to 0.2 kb downstream of transcription start site (Figure 1A). Details regarding primers, re‐sequencing procedures and data analysis are given in Table S1 and the Methods section in the supplemental materials.

### 2.3 In silico analysis of RIP3 promoter region

A total of 14 common single nuclear polymorphism (SNPs; Minor allele frequency [MAF] > 0.01) in RIP3 were identified by Sanger sequencing. The respective regions flanking these variants were analysed for potential transcription factor binding sites using Jaspar (http://jaspar.genereg.net/).^22^

### 2.4 SNP selection and genotyping

Genomic DNA was extracted from peripheral leucocytes as previously reported.^23^ ABI Primer Expression 3.0 software was used for design of probe and primer sequences, which were synthesized by Shanghai GeneCore Bio Technologies Co., Ltd, China. The common variants in RIP3 promoter and CDS regions were genotyped using the TaqMan assay on the TaqMan 7900HT Sequence Detection System (Applied Biosystems, Foster City, CA) with the following conditions: 10 minutes at 95°C (enzyme activation) followed by 45 cycles at 95°C for 15 seconds and 60°C for 1 minute (annealing/extension). An endpoint read was performed for allelic discrimination after amplification. The details of the procedure for amplification and the quality control of genotyping were mentioned in our previous study.^24^ Details regarding primers and probes can be found in Table S2.

### 2.5 Plasmids construction, cell culture, transient transfection and luciferase activity assays

To determine whether rs3212247 and other seven polymorphisms are in strict linkage disequilibrium with its influence transcription activity of RIP3 gene, we constructed 14 reporter plasmids including their wild and mutant type using PGL3‐basic. Meanwhile, nine transcriptional factors include SOX17, PBX1, SRY, ZNF35, TEAD4, ZNF354C, SOX9, SOX10 and SOX21 were cloned into pcDNA3.1 using human cDNA. Flag‐tagging was constructed into the 5' of SOX17 initiation codon. AC16, a cell line derived from adult human ventricular cardiomyocyte,^25^ which can well exhibit ultrastructural, molecular genetics and immunocytochemical characteristics of cardiomyocyte, has been used to investigate development regulation of cardiomyocyte as an in vitro model.^26^, ^27^ Thus we used AC16 and HEK293T cell to conduct luciferase reporter assay. Cell culture and transient transfection procedures are described in detail in the supplemental materials. Cells were harvested 36 to 48 hours after transfection using the Passive Lysis Buffer (SIRIUS, Pforzheim, Germany). The data of luciferase expression levels were adjusted with reference to Renilla luciferase activity and relative to the average values of wild‐type for corresponding variants. Each reporter was performed six independent experiments to avoid potential experimental errors.

### 2.6 Western blotting for RIP3

Cells grown in 6‐well plates were transfected with SOX17 constructs or empty vector pcDNA3.1 using Lipofectamine™ 2000 transfection reagent (Invitrogen) according to the manufacturer's instructions. Cells were harvested 48 hours after transfection and lysed with lysis solution (50 mmol/L Tris‐Cl, pH 8.0; 150 mmol/L NaCl; 0.02% sodium azide; 0.1% SDS; 1 μg/mL aprotinin; 1% Nonidet P‐40; and 0.5% sodium deoxycholate) containing protease inhibitors (100 μg/mL phenylmethylsulfonyl fluoride, 2 μg/mL aprotinin, 2 μg/mL leupeptin). After centrifuging at 12,000 *g* for 20 minutes at 4°C, supernatant was collected and protein concentrations were measured using the BCA protein assay reagent kit (Boster, China). Lysates were resolved by 10% SDS‐PAGE and transferred to polyvinylidene difluoride membranes. After blocking with 5% non‐fat milk, blots were probed with RIP3 antibody (Abcam, ab56164) and incubated with a peroxidase‐conjugated secondary antibody. Bands were visualized by enhanced chemiluminescence reagents (Pierce Chemical, Rockford, IL) and quantified by densitometry.

### 2.7 Chromatin immunoprecipitation assay

Chromatin immunoprecipitation assays were carried out using commercially available assay kits. Detailed descriptions are available in supplemental materials.

### 2.8 Electrophoretic mobility‐shift assay

Detailed procedures performed in the study are described in the supplemental materials.

### 2.9 Measurement of genotype‐dependent plasma levels of RIP3

Sex, age and NYHA class matched 207 HF patients and 78 sex, age‐matched controls were selected to detect the plasma levels of RIP3. The detailed clinical characteristics and biochemical profiles of the samples are shown in Table S3. Total plasma RIP3 levels were analysed using a Human RIP3 ELISA Kit (CUSABIO, Wuhan, China) according to the manufacturer's instructions.

### 2.10 Statistical analyses

Statistical analyses were performed with spss version 13.0 (SPSS, Inc, Chicago, Illinois) for Windows (Microsoft Corp, Redmond, Wash). Linkage disequilibrium was calculated using Haploview version 4.1. The polymorphisms were tested for Hardy‐Weinberg equilibrium among the HF patients and the controls using χ^2^ test. We performed multivariate logistic regression analyses based on the different genetic models with adjustment of traditional risk factors to test the association between the SNPs and HF. Clinic prognosis analysis for HF was performed with Cox proportional hazards regression model with or without adjustment for traditional risk factors. Significant differences were assessed by either one‐way ANOVA followed by Bonferroni's post‐hoc test or unpaired or paired, two‐tailed Student's *t* test, where appropriate. Because the distribution of RIP3 plasma levels is skewed non‐normally, log transformation was performed and the results fitted normal distribution.

All biostatistics calculations were performed with Prism (GraphPad). Data are expressed as mean ± SEM of n experiments. All probability values were two‐sided and *P* < 0.05 was considered to be significant.

## 3 RESULTS

### 3.1 DNA re‐sequencing results, haplotype analysis of common variants within RIP3 gene and identification of candidate functional SNPs

We identified 14 common polymorphisms (MAF > 0.01) in the promoter and 5'UTR region of RIP3 by re‐sequencing 200 randomly selected unrelated healthy individuals from the Han Chinese population, including 12 in promoter and two in 5'UTR (Table 2). All the 14 polymorphisms above were in Hardy‐Weinberg equilibrium in our sample (*P* > 0.05). Our re‐sequencing results were in consistent with the Chinese data from the 1000 Genomes Browser, in which we found only one common missense variant rs3212254 in the coding regions but none in the 3'UTR. Referring to the Chinese data of the 1000 Genomes, rs3212254 was in strong linkage disequilibrium with rs3212241 and rs3212245 (*r* ^2^ = 1). Thus combining the genotypes of 200 healthy controls above and Chinese data from the 1000 Genomes Browser, we identified 4 haploblock structures within RIP3 gene in the Han Chinese population (Table S4). Considering a substantial of variants in RIP3, we attempted to narrow down candidate functional SNPs, through which the causal SNP might be precisely and rapidly found out. RegulomeDB (http://www.regulomedb.org/index) is a database that contains experimental data from ENCODE and other sources in addition to computational predictions of regulatory potential and annotates SNPs with known and predicted regulatory elements in the intergenic regions of *H. sapiens* genome.^28^ RegulomeDB assigns each SNP a score and 1 represents probably functional, 6 represents unlikely functional. Adam et al have successfully identified the functional variant associated with coronary artery disease using RegulomeDB database.^29^ Therefore we looked up all the 14 common SNPs in the RegulomeDB database to localize truly functional variant. The result showed that rs3212249 represented a suitable candidate functional variant (Table S4). Considering that rs3212249 was in strong linkage disequilibrium with other seven variants (Figure S1), we performed further functional studies of all the eight polymorphisms (include rs3759630, rs3212245, rs3212246, rs3212247, rs3759625, rs3834521, rs3212250, rs3212251). The variant rs3212254 was in coding region of RIP3 and was predicted to be probably damaging as predicted at PolyPhen‐2 (http://genetics.bwh.harvard.edu/pph2/), and thus, we also genotyped rs3212254 in control and HF groups.

**Table 2:** Characteristics of RIP3 variants identified by Sanger sequencing

Gene Position^a^	dbSNP ID^b^	Gene Region	Maj>Min^c^	MAF
chr14:24810898	rs3212240	promoter	G/A	0.163
chr14:24810830	c.‐1798A>G	promoter	G/A	0.005
chr14:24810636	c.‐1604A>G	promoter	G/A	0.003
chr14:24810438	rs3212241	promoter	C/T	0.138
chr14:24810413	rs3759630	promoter	G/A	0.295
chr14:24810401	rs3212242	promoter	G/A	0.268
chr14:24810370	rs3212243	promoter	A/G	0.268
chr14:24810336	c.‐1304A>G	promoter	C/T	0.003
chr14:24810248	c.‐1216A>G	promoter	C/T	0.003
chr14:24809957	rs3212245	promoter	T/A	0.135
chr14:24809850	rs3212246	promoter	G/T	0.29
chr14:24809795	rs3212247	promoter	T/C	0.29
chr14:24809683	rs3759625	promoter	T/C	0.29
chr14:24809650	rs3834521	promoter	‐/C	0.287
chr14:24809611	rs1007381	promoter	C/T	0.163
chr14:24809415	rs3212249	promoter	G/A	0.287
chr14:24809191	rs3212250	5′UTR	A/G	0.287
chr14:24809185	rs3212251	5′UTR	A/G	0.287

Abbreviations: MAF, Minor allele frequency; RIP3, Receptor‐interacting protein kinase 3.

a Base pair position is based on NCBI GRCH37.

b Polymorphisms are numbered relative to transcription start site.

c With major allele given first, followed by minor allele.

### 3.2 Effects of polymorphisms on the transcriptional activity

Firstly, we performed functional analyses comparing the activities of the ‘major’ (Hap1) and ‘minor’ (Hap2) haplotype in AC16 cells. As shown in Figure 1B, firefly luciferase reporter gene expression of the minor haplotype increased significantly compared to the major haplotype. To identify which was the causal variant, all the eight SNP‐oriented reporter plasmids were constructed and transfected into AC16 cells. The results showed that reporter gene expression of the rs3212247‐C allele was significantly increased compared with the rs3212247‐T allele (Figure 1C), which could be replicated in HEK293T cell (Figure S2A). However, we did not observe any effect on luciferase activity in rs3834521, rs3759625, rs3212249, rs3759630 and rs3212246 luciferase assays in AC16 cells (Figure 1D‐H), expect that the wild‐type of haplotype, which contains rs3212250 and rs3212251, showed higher reporter gene expression than mutant type (Figure 1I). These results indicate that rs3212247 in the promoter of RIP3 gene may affect the binding domain of an endogenous cardiac regulator factor and in turn affect the gene expression.

### 3.3 SOX17 modulates RIP3 expression

**Figure 2:**
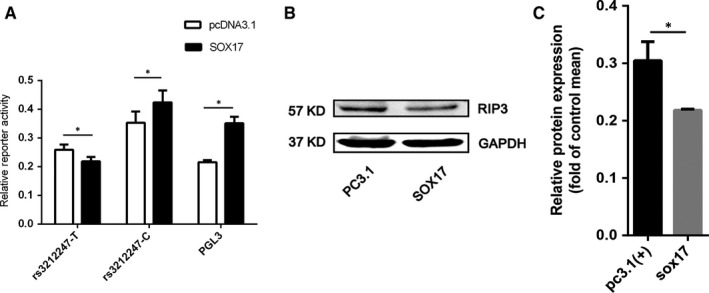
SOX17 binds to the rs3212247‐T allele and mediates allelic expression. (A), Firefly luciferase assays were performed with 250‐bp rs3212247‐T or ‐C allele constructs cotransfected into AC16 cells with SOX17 vector encoding sox17. Luciferase activity was measured 48 h after transfection and was normalized against Renilla luciferase activity. (B), AC16 cells were transfected with pcDNA3.1 and SOX17 respectively. Protein was harvested 48 h after transfection and Receptor‐interacting protein kinase 3 (RIP3) expression was normalized against GAPDH. *P < 0.05

Bioinformatics analysis indicated that rs3212247 located in the binding site of SOX17, PBX1, SRY, ZNF35, TEAD4, ZNF354C, SOX9, SOX10 and SOX21. In order to confirm the effects of all these transcription factors (TFs) on endogenous RIP3 expression, we sequenced AC16 and HEK293T cell lines and found them to be TT genotype (Figure S3). Subsequently, AC16 and HEK293T cells were transfected with pcDNA3.1 and TFs respectively. Western blot results revealed that SOX17 down‐regulated RIP3 expression in both AC16 (Figure 2B) and HEK293T cells (Figure S2B). No changes were observed in RIP3 expression for other eight TFs in AC16 cells (Figure S4). We then experimentally investigated the effect of rs3212247 on SOX17‐mediated gene expression using Luciferase Activity Assays. Rs3212247 promoter reporter plasmids were cotransfected with SOX17 into AC16 cells. As shown in Figure 2A, compared with PGL3 empty vector, transcription activity of rs3212247‐T allele reduced after transfected with SOX17 expression plasmids, which was in line with previous report that SOX17 could act as an inhibitory transcription factors.^30^ Meanwhile, rs3212247‐T allele exhibited lower firefly luciferase activity compared with rs3212247‐C allele after transfected with SOX17. Consistent result was observed in HEK293T (Figure S2A). This indicated that the rs3212247‐C allele could disrupt the binding site of SOX17 in the RIP3 promoter and increased the transcriptional activity of the gene.

### 3.4 In vivo and vitro analyses of SOX17 binding for the RIP3 rs3212247‐T>C allele

**Figure 3:**
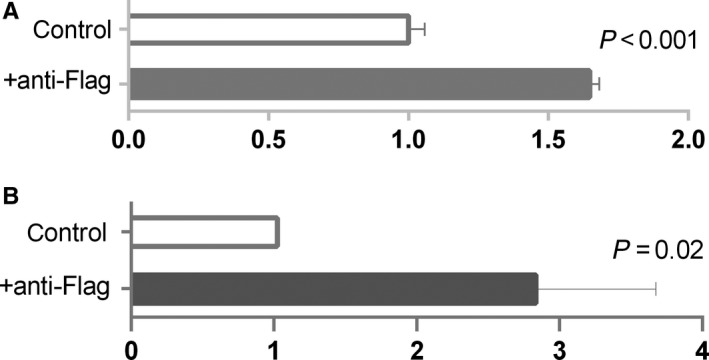
Chromatin immunoprecipitation with antibody against Flag‐SOX17 in AC16 (A) and HEK293T (B) with transduction with Flag‐SOX17 construct. Quantitative PCR was used to measure the Immunoprecipitation of DNA sequence surrounding rs3212247, normalized to background (control condition with IgG, no antibody)

In order to further assess the functional relevance of rs3212247, we performed DNA binding analysis in AC16 and HEK293 cells. Chromatin Immunoprecipitation Assay was conducted and the results showed that SOX17 binding to the region encompassing rs3212247 was detected in both cell lines (Figure 3).

**Figure 4:**
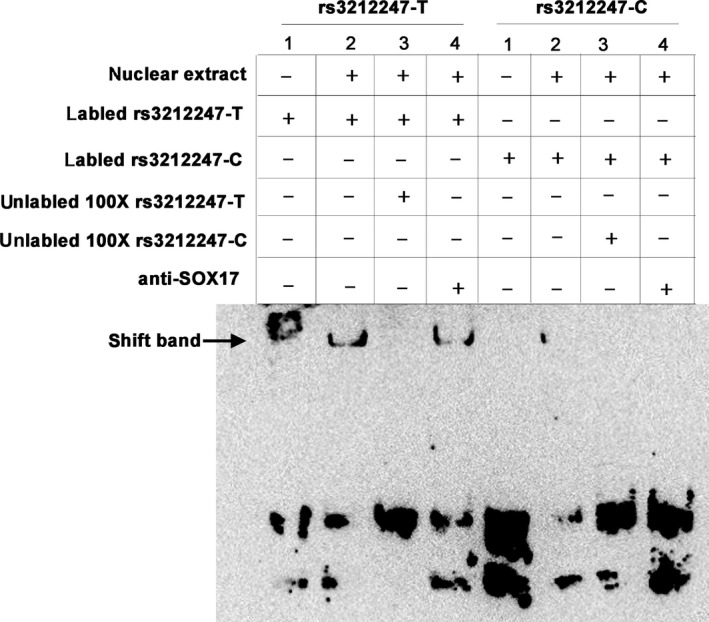
In vitro SOX17 binding to rs3212247‐T allele. Gel‐shift assay was performed with a biotin‐labelled probe containing SOX17 binding elements with nuclear extract from AC16 or absence of nuclear extract in each group (lane 1), without or with competition from unlabelled oligonucleotides containing rs3212247‐T or rs3212247‐C ins probes (lanes 2 to 3 respectively) or anti‐SOX17 antibody as indicated (lane 4)

Meanwhile, we carried out electrophoretic mobility‐shift assays in AC16. As shown in Figure 4, SOX17‐containing nuclear extracts prefer to bind to biotin‐labelled rs3212247‐T probe but not to rs3212247‐C probe and additional unlabelled oligonucleotide partially competed with this binding. However, no supershift band was present.

### 3.5 Association of rs3212247 with plasma RIP3 levels

**Figure 5:**
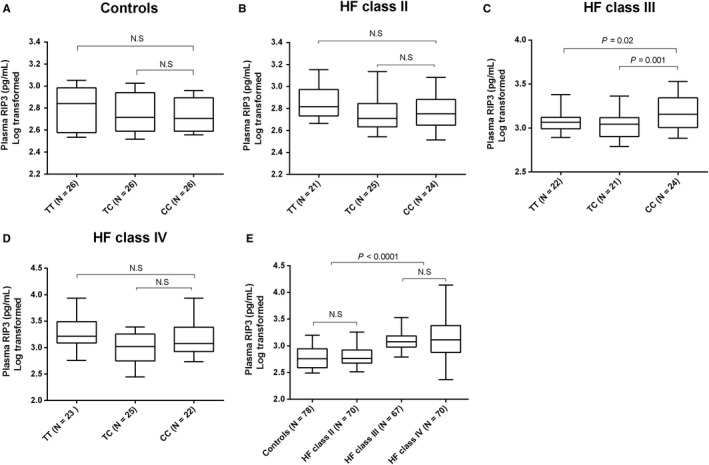
In vivo plasma concentrations of Receptor‐interacting protein kinase 3 (RIP3) in control group (A) HF NYHA class II group (B) HF NYHA class III group (C) HF NYHA class IV group (D) and among these four groups (E). The data are presented as box (25th percentile, median and 75th percentile)

We measured the plasma RIP3 concentrations of HF patients to further investigate the functional role of rs3212247. As shown in Figure 5, plasma concentrations of RIP3 were higher in individuals with the CC genotype than in those with TT or TC genotypes among the NYHA class III patients (p for trend = 0.02). However, we did not observe the same results in other groups including NYHA II, NYHA IV and control groups. Interestingly, the plasma RIP3 levels of NYHA III and IV groups were significantly higher compared with NYHA II and control groups, suggesting that higher RIP3 levels may aggravate HF.

Furthermore, we assessed the effect of β‐blocker use on plasma RIP3 level. No difference was observed between HF patients with and without β‐blocker use in NYHA II, NYHA III, NYHA IV and combined groups (Figure S5).

### 3.6 Association of rs3212247 with the risk and prognosis of HF

**Figure 6:**
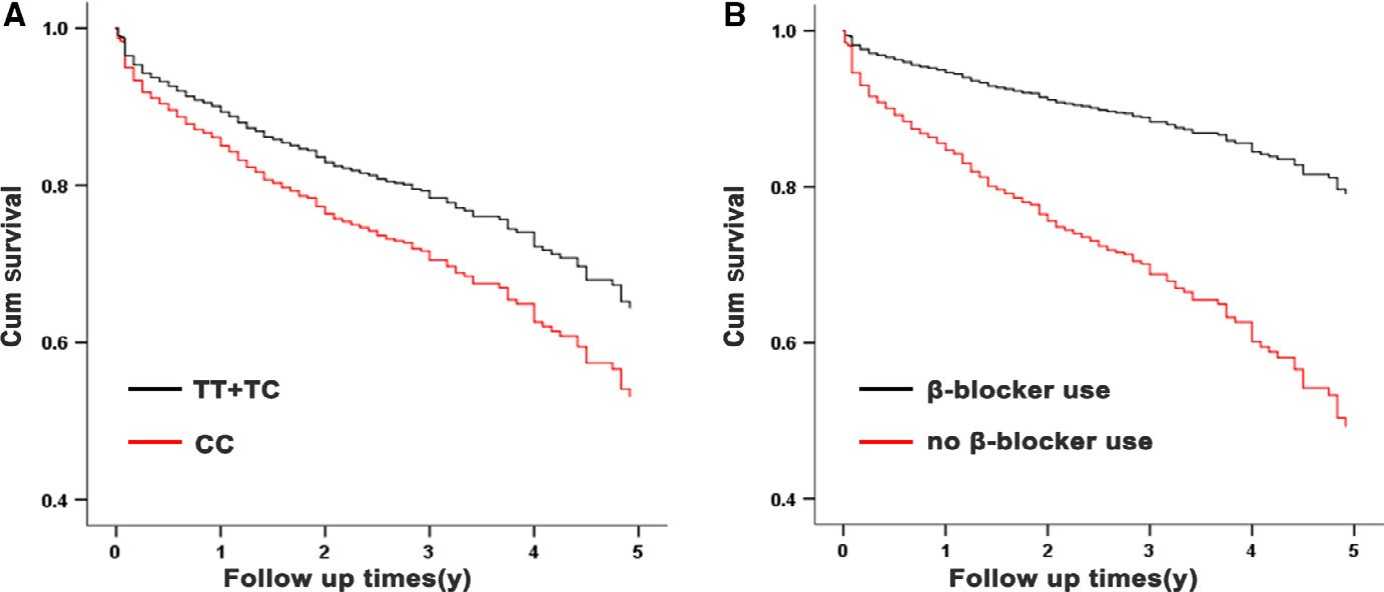
Effects of rs3212247 on the prognosis of heart failure (HF) patients. (A), Cox proportional hazards models analysis showed the association of genotypes of rs3212247 with cardiovascular deaths or cardiac transplantation (unadjusted HR = 1.47, 95% CI = 1.13‐1.91; *P* = 0.004). (B), Comparison of 3194 HF patients with and without β‐blocker use (adjusted HR = 0.36, 95% CI = 0.29‐0.43; *P* < 0.001)

Since it affected the expression and plasma concentration of RIP3, we further tested the association of rs3212247 with risk and prognosis of HF. Considering that rs3212247 was in strong linkage disequilibrium with rs3212250 and rs3212251 (*r* ^2^ = 0.99), we chose rs3212250 combined with rs3212251 as a tagging SNP for genotyping because they were only 6‐bp distance. As a result, no significant difference was observed in genotype of rs3212247 between cases and controls (Table 3), which indicated that this SNP is not associated with the risk of HF. Interestingly, rs3212247 was significantly associated with the prognosis of HF using genetic recessive model, both with or without adjustment for conventional risk factors (including sex, age, hypertension, diabetes, hyperlipidemia, smoking state) and β‐blocker use (Figure 6A). The cardiovascular death or cardiac transplantation had occurred in 261 patients (16.4%) in TT genotype group, 201 patients (15.5%) in TC genotype group and 65 patients (21.5%) in CC genotype group. Cox proportional hazards models analysis showed that the C allele of rs3212247 is significantly associated with an increased risk of cardiovascular death and cardiac transplantation (hazard ratio [HR] = 1.47, 95% CI = 1.13‐1.91; *P* = 0.004). The statistical significance in multivariate analysis remained after adjustments for sex, age, hypertension, diabetes, hyperlipidemia, smoking state and β‐blocker using (HR = 1.40, 95% CI = 1.06‐1.84; *P* = 0.018) (Table 4). No statistical significance was observed using additional and dominant model (Table 4). Meanwhile, we observed no association between rs3212254 genotypes and the risk or prognosis of HF in case‐control or case‐only study after adjustment for traditional risk factors (Tables 3 and 4).

**Table 3:** Association between RIP3 variants and heart failure

SNP rs ID	Function	Population	MAF	Genotype			Model	Crude ORs	Adjusted	Adjusted ORs
								(95%CI)	*P*‐Value	(95%CI)
				TT	TC	CC	Additive	1.00 (0.92‐1.08)	0.53	1.03 (0.94‐1.14)
rs3212247	Promoter	Control	0.3	1454	1243	264	Dominant	0.97 (0.88‐1.07)	0.99	1.00 (0.88‐1.14)
T > C		HF	0.3	1593	1298	303	Recessive	1.07 (0.90‐1.27)	0.16	1.17 (0.94‐1.46)
				GG	GT	TT	Additive	1.08 (0.97‐1.20)	0.46	1.06 (0.92‐1.22)
rs3212254	CDS	Control	0.12	2270	658	33	Dominant	1.04 (0.93‐1.17)	0.67	0.97 (0.85‐1.11)
G > T		HF	0.13	2424	708	62	Recessive	1.76 (1.15‐2.69)	0.18	1.45 (0.84‐2.51)

Abbreviations: HF, heart failure; MAF, Minor allele frequency; RIP3, Receptor‐interacting protein kinase 3; SNP, single nuclear polymorphism.

Odds Ratios (ORs) and 95% confidence intervals (CIs) were obtained by logistic regression, with and without adjustment for sex, age, hypertension, diabetes, hyperlipidemia and smoking status.

**Table 4:** Association of the RIP3 polymorphisms with cardiac mortality or transplantation in chronic heart failure

	Dominant model		Recessive model		Additive model	
SNPs	HR (95% CI)	*P*	HR (95% CI)	*P*	HR (95% CI)	*P*
rs3212247						
Unadjusted	0.97 (0.81‐1.15)	0.68	1.47 (1.13‐1.91)	0.004*	1.11 (0.98‐1.27)	0.11
Adjusted	0.95 (0.80‐1.14)	0.61	1.40 (1.06‐1.84)	0.018*	1.10 (0.96‐1.27)	0.15
rs3212254						
Unadjusted	1.04 (0.85‐1.27)	0.69	1.18 (0.61‐2.28)	0.62	1.02 (0.85‐1.22)	0.83
Adjusted	0.93 (0.75‐1.15)	0.49	1.00 (0.52‐1.94)	1	1.06 (0.88‐1.28)	0.54

Hazard Ratio (HR) and 95% confidence intervals (95% CI) were obtained by Cox regression, without and with adjustment for sex, age, hypertension, diabetes, hyperlipidemia, smoking status and β‐blocker use.

Abbreviations: RIP3, Receptor‐interacting protein kinase 3; SNP, single‐nucleotide polymorphism.

*P < 0.05.

**Figure 7:**
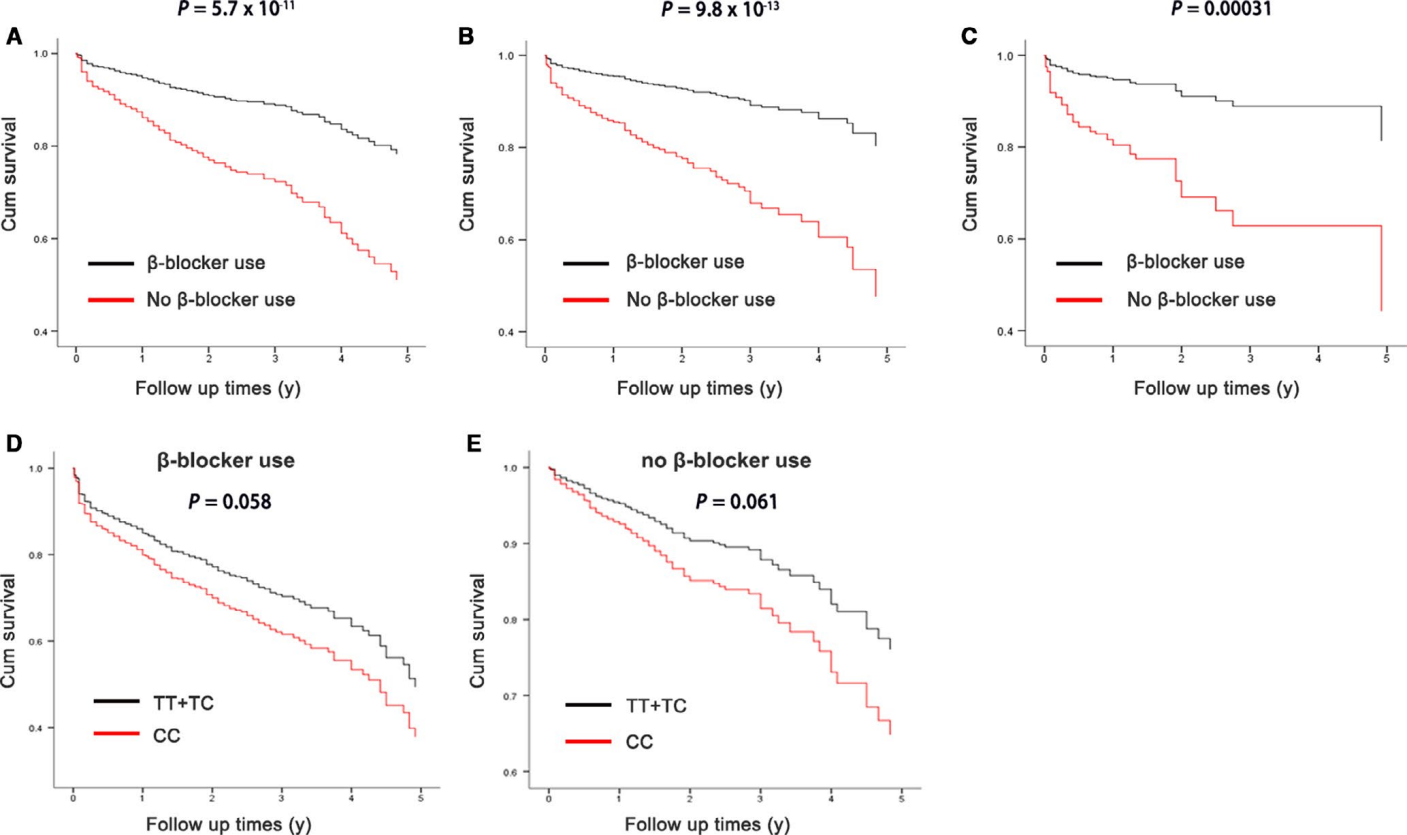
Prospective analysis of the interaction between rs3212247 and β‐blocker use as a determinant of heart failure. Cox proportional hazards model was used for comparison of rs3212247‐TT genotype (A) rs3212247‐TC genotype (B) and rs3212247‐CC genotype (C) with and without β‐blocker use, respectively. (D, E), Comparison of different genotype when stratified by β‐blocker use

Furthermore, we analysed the effects of β‐blocker treatment on the prognosis of HF in all patients and stratified by genotype of rs3212247. Consistent with findings from previous clinical studies,^31^, ^32^ β‐blocker treatment was associated with a reduced risk of cardiac transplantation or deaths (adjust HR = 0.36, 95% CI = 0.29‐0.43; *P* < 0.001) (Figure 6B). The benefits of β‐blocker treatment were significant stratified by rs3212247 genotypes after multivariate analysis (adjusted HR = 0.40; 95% CI = 0.31‐0.53; *P* < 0.001 in the TT group; adjusted HR = 0.32; 95% CI = 0.23‐0.44; *P* < 0.001 in the TC group; and adjusted HR = 0.33; 95% CI = 0.18‐0.59; *P* < 0.001 in the CC group) (Figure 7A‐C). And the effect of genotype on prognosis of HF has no statistical significance but showed trend after stratified by β‐blocker use (Figure 7D‐E).

## 4 DISCUSSION

**Figure 8:**
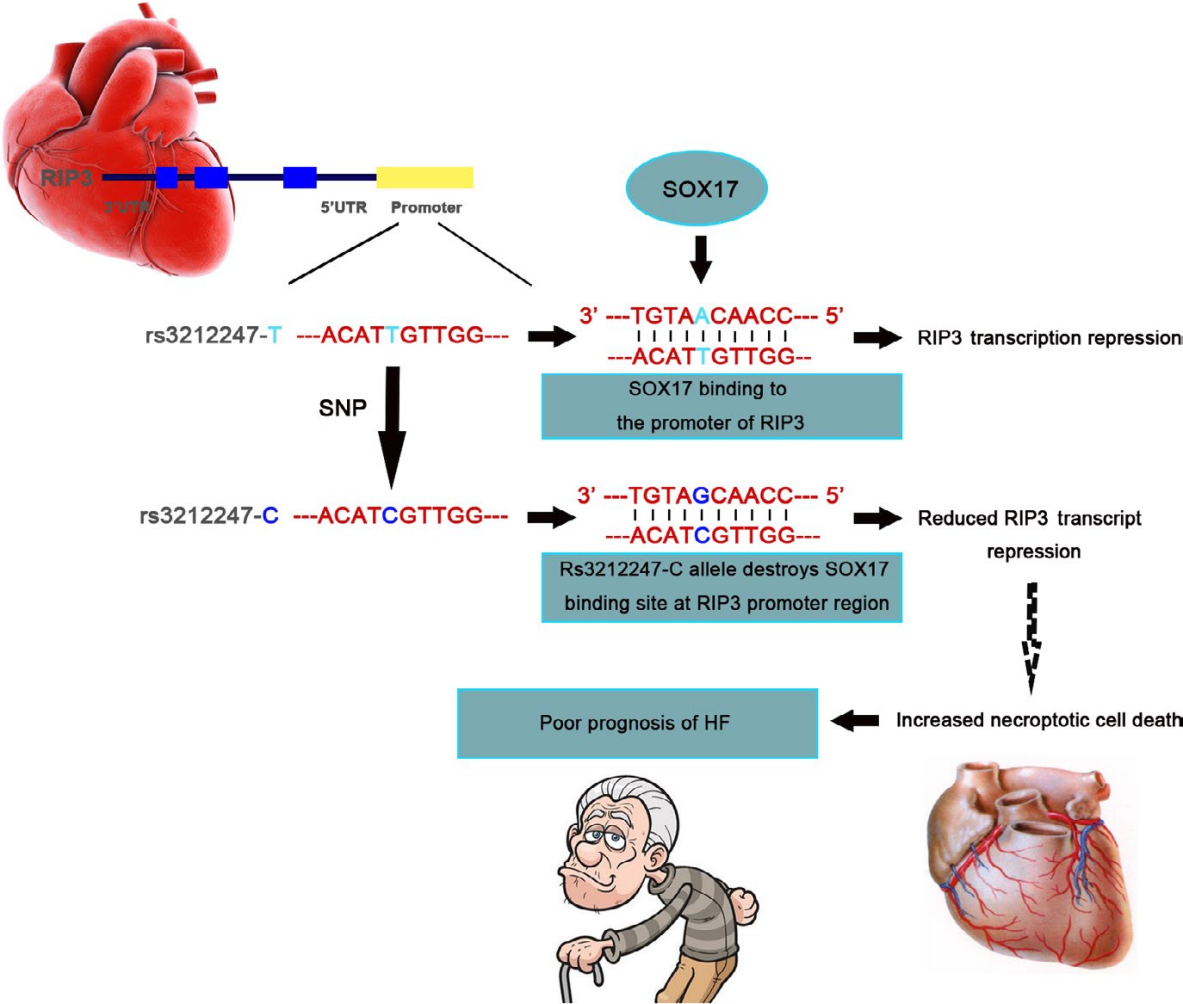
Effect of SOX17 on Regulation of Receptor‐interacting protein kinase 3 (RIP3) Transcription. The variant rs3212247 in RIP3 promoter region is a T‐ to‐C change and is predicted to locate in the binding site of SOX17. Rs3212247‐C allele destroys the SOX17 binding site in the promoter region of RIP3 and subsequently results in increased transcription and translation of RIP3, followed by increased necroptosis of cardiomyocyte under various external stimuli, which leads to poor prognosis of heart failure (HF)

Heart failure is a common chronic disorder accompanied by loss of terminally differentiated cardiomyocyte.^21^ As a passive and unregulated death form, necrosis has long been considered as the main form of cell death in myocardial injury.^33^, ^34^ Emerging evidence has shown that RIP3 participated in necroptosis, which is a programmed necrosis regulated by RIP1‐RIP3‐dependent death signalling pathway.^21^, ^35^ In heart, RIP3 enrolled in I/R and MI‐induced myocardial injury and remodelling, which could be ameliorated by RIP3 knockout.^18^, ^21^ Considering the important role of RIP3 in myocardium, we investigated the association of single‐nucleotide polymorphisms in RIP3 gene with HF using case‐control and case‐only studies involving 3194 HF patients and 2961 control participants. Our results demonstrated that rs3212247 in RIP3 promoter region was significantly associated with the prognosis of HF (HR = 1.47, 95% CI = 1.13‐1.91; *P* = 0.004). The statistical significance remained after adjustment for conventional cardiovascular risk factors (sex, age, hypertension, diabetes, hyperlipidemia and smoking state) and β‐blocker use, suggesting that the effect of this polymorphism on the prognosis of HF is independent of current known risk factors. No association was found between rs3212247 and risk of HF. On the contrary, β‐blocker could significantly improve the survival of HF patients and the benefits were independent of genotypes of rs3212247. After stratified by β‐blocker use, no statistical significance but trend was observed when analysing the effect of different genotype on prognosis of HF, which may be attributed to insufficient population of rs3212247‐CC genotype. Furthermore, β‐blocker had no effect on plasma RIP3 level. All these population‐based results indicated no association between RIP3 and β‐adrenergic receptor signalling. Functional analyses showed that rs3212247‐C allele disrupted the binding site of transcriptional factor SOX17, an inhibitory transcriptional factor, and therefore increased the transcriptional activities of RIP3. Moreover, in NYHA class III HF group, the plasma RIP3 levels of rs3212247‐C homozygote carriers were significantly higher than those of the rs3212247‐T carriers. Even so, consistent results were not observed in other groups (including NYHA class II, IV and control groups). This inconsistence may be attributed to the wide expression of RIP3 in colon, spleen and lung,^19^ which needs to be further investigated in a larger population. Interestingly, plasma concentration of RIP3 of NYHA III and IV patients was significantly higher than that of NYHA II and control individuals, which revealed that RIP3 may contribute to exacerbation of HF. However, Javor et al found no difference of serum levels of RIP3 between NYHA class III‐IV patients and healthy individuals, which may be attributed to insufficient samples.^36^ Taking together, our findings indicated that rs3212247 could modify the progress of HF through disrupting the binding efficiency of SOX17 and reducing the expression of RIP3 (Figure 8).

In our study, the CC genotype of rs3212247 was resistant to SOX17‐induced down‐regulation of RIP3 and resulted in higher RIP3 expression, which could further lead to poorer prognosis of HF patients. So far, substantial studies have reported that SOX17 is a tumour suppressor in many kinds of tumours including endometrial cancer, cholangiocarcinoma, oesophageal cancer, et al^30^, ^37^, ^38^ Moreover, SOX17 is indispensable for the specification of cardiac mesoderm and participates in cardiac differentiation,^39^, ^40^ indicating a pivotal role in myocardium. In the present study, we found that SOX17 overexpression reduced the expression of RIP3 in AC16 cell, which indicated that SOX17 might implicate in the regulation of necroptosis of cardiomyocyte. However, up to now, no investigation was conducted to explore the role of genetic variants in RIP3 in HF. Cerhan et al^41^ have performed clinic‐based case‐control study using the Affymetrix Immune and Inflammation SNP panel which revealed the association of variants in RIP3 gene with risk of Non‐Hodgkin lymphoma. A rare variant in MLKL, as a functional substrate of RIP3,^42^ was found to be associated with risk of late‐onset AD.^43^ Thus, our study firstly demonstrated the association of variant in RIP3 gene with the prognosis of HF.

However, there exist some limitations of our study. Firstly, based on our re‐sequencing results only the variants of RIP3 gene were investigated. Other functional variants in nearby genes, which were in strict linkage disequilibrium with rs3212247 may also participate in the prognosis of HF. This needs to be further investigated in other studies. Secondly, since this investigation was conducted in only one population, future studies need to be replicated in another geographically unrelated population. Furthermore, other transcriptional factors may involve in the regulation of RIP3 gene expression.

In conclusion, our results provide strong evidence that rs3212247 in promoter region of RIP3 was associated with the prognosis of HF. The rs3212247‐C allele may destroy the binding site of repressor transcriptional factor SOX17, thus increase the transcription of RIP3 and further lead to poorer prognosis of HF. Moreover, the high hazard ratios (HR = 1.47, 95% CI = 1.13‐1.91; *P* = 0.004) and high frequency of the risk allele (MAF = 0.3) indicated that this variant may be responsible for a substantial proportion of poor prognosis of HF patients. Further prevention and treatment strategies through targeting RIP3 is an attractive way to decrease HF associated mortality and improve the prognosis of HF.

## CONFLICT OF INTEREST

The authors declare no competing financial interests.

## AUTHOR CONTRIBUTIONS

Dong Hu and Jin Huang developed the study concept, design and interpreted the data and drafted the manuscript. Senlin Hu, Ying Zhang and Shiyang Li performed the experiments, analysed the data. Yang Sun, Chenze Li, Guanglin Cui, Daowen Wang supervised the design of the study and revised the manuscript.
