# Supplementary figures and images for: A common variant of RIP3 promoter region is associated with poor prognosis in heart failure patients by influencing SOX17 binding
Source: J Cell Mol Med. 2019 May 31;23(8):5317–28. doi: 10.1111/jcmm.14408 (PMC6652837; doi:10.1111/jcmm.14408)

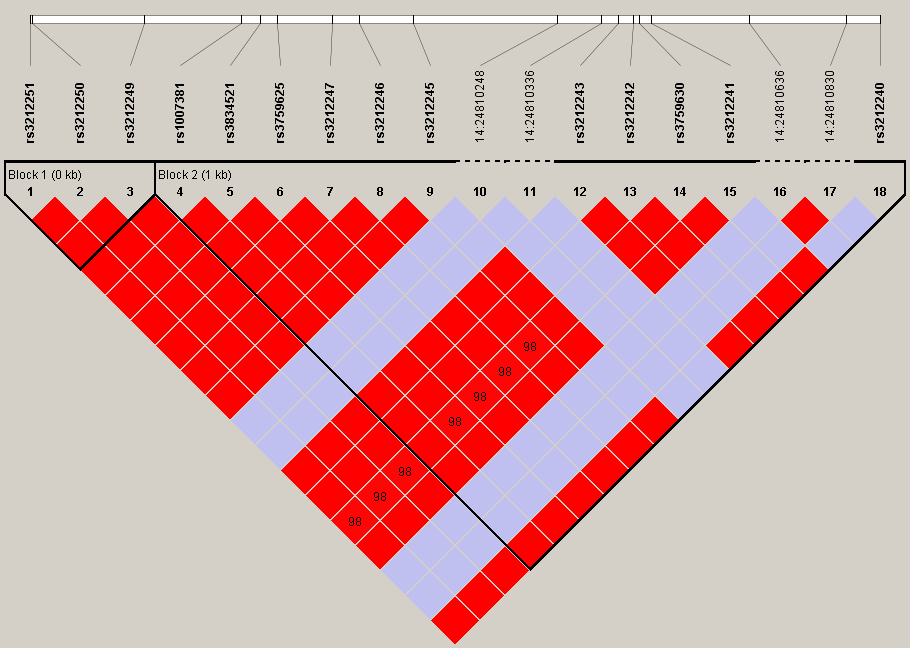

Supplement: Supplementary file 1 [file JCMM-23-5317-s001.tif]

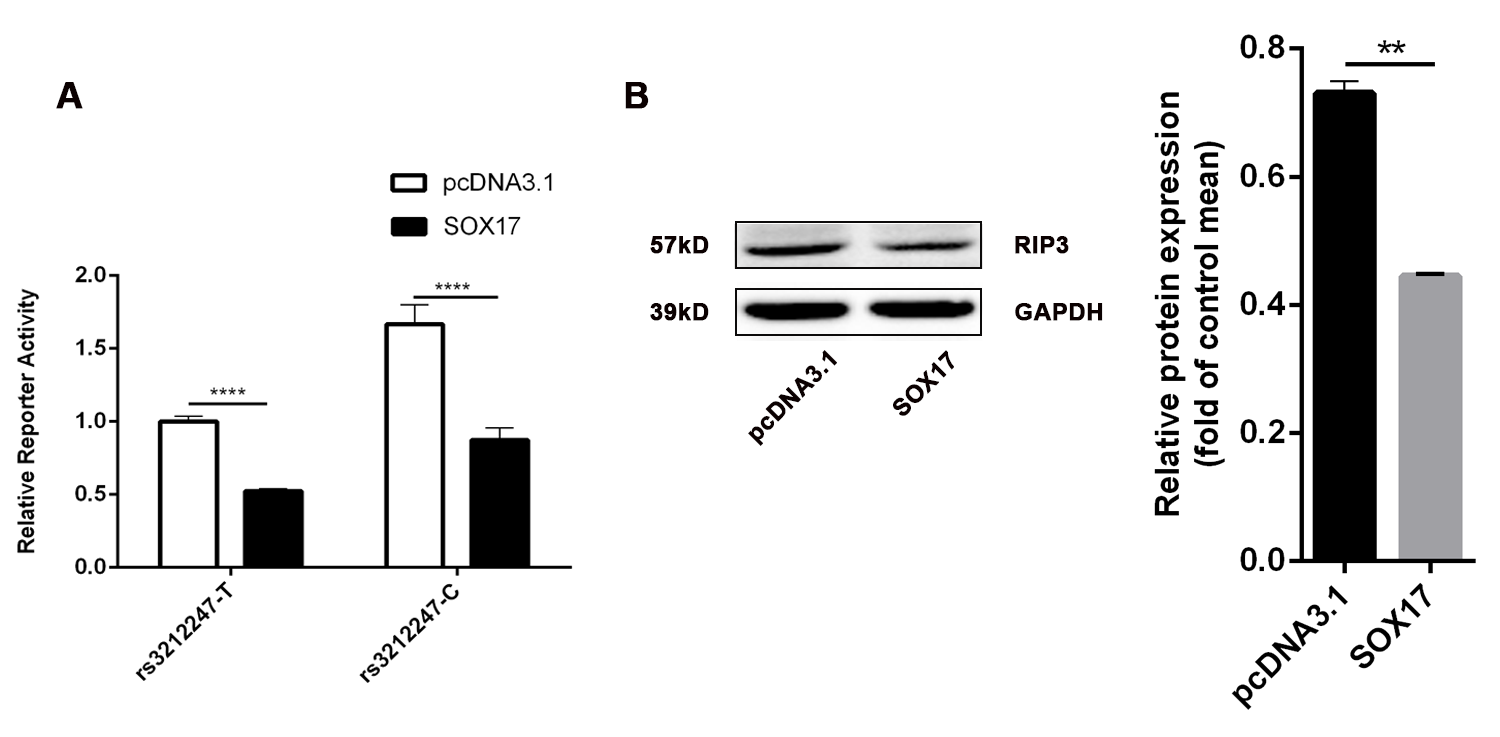

Supplement: Supplementary file 2 [file JCMM-23-5317-s002.tif]

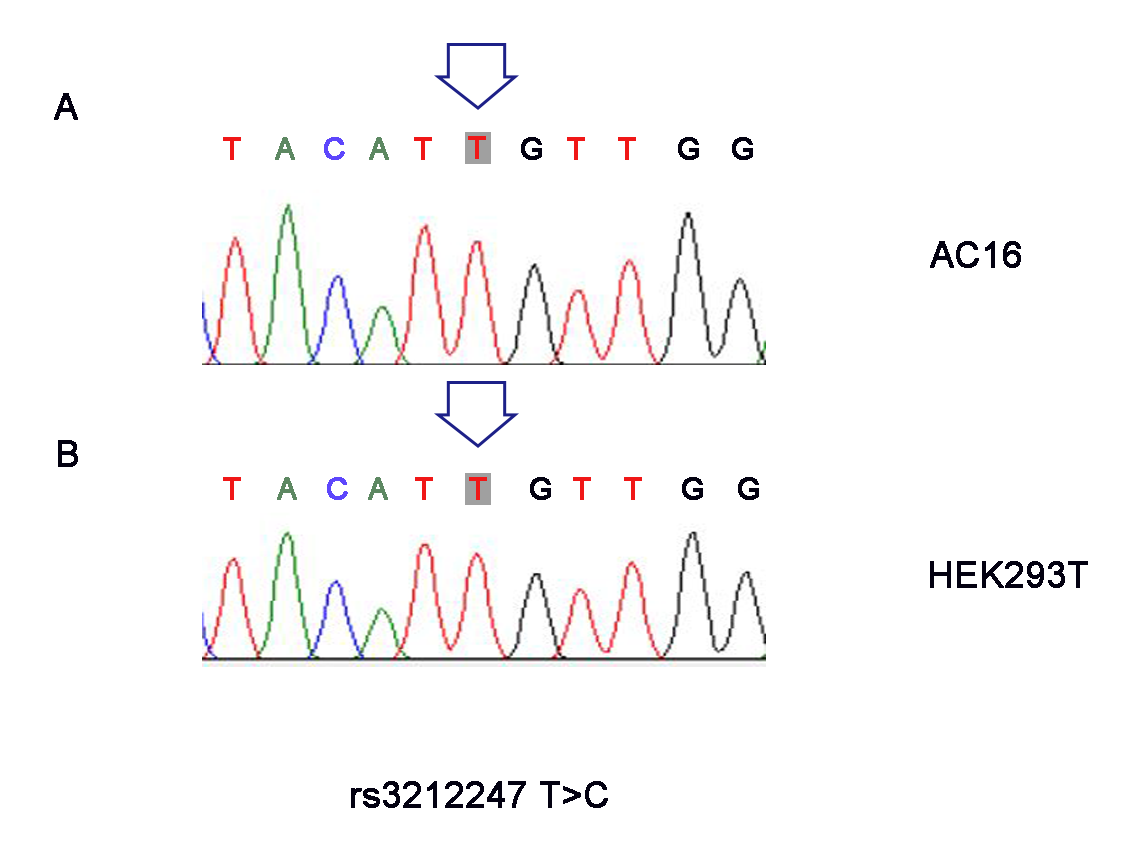

Supplement: Supplementary file 3 [file JCMM-23-5317-s003.tif]

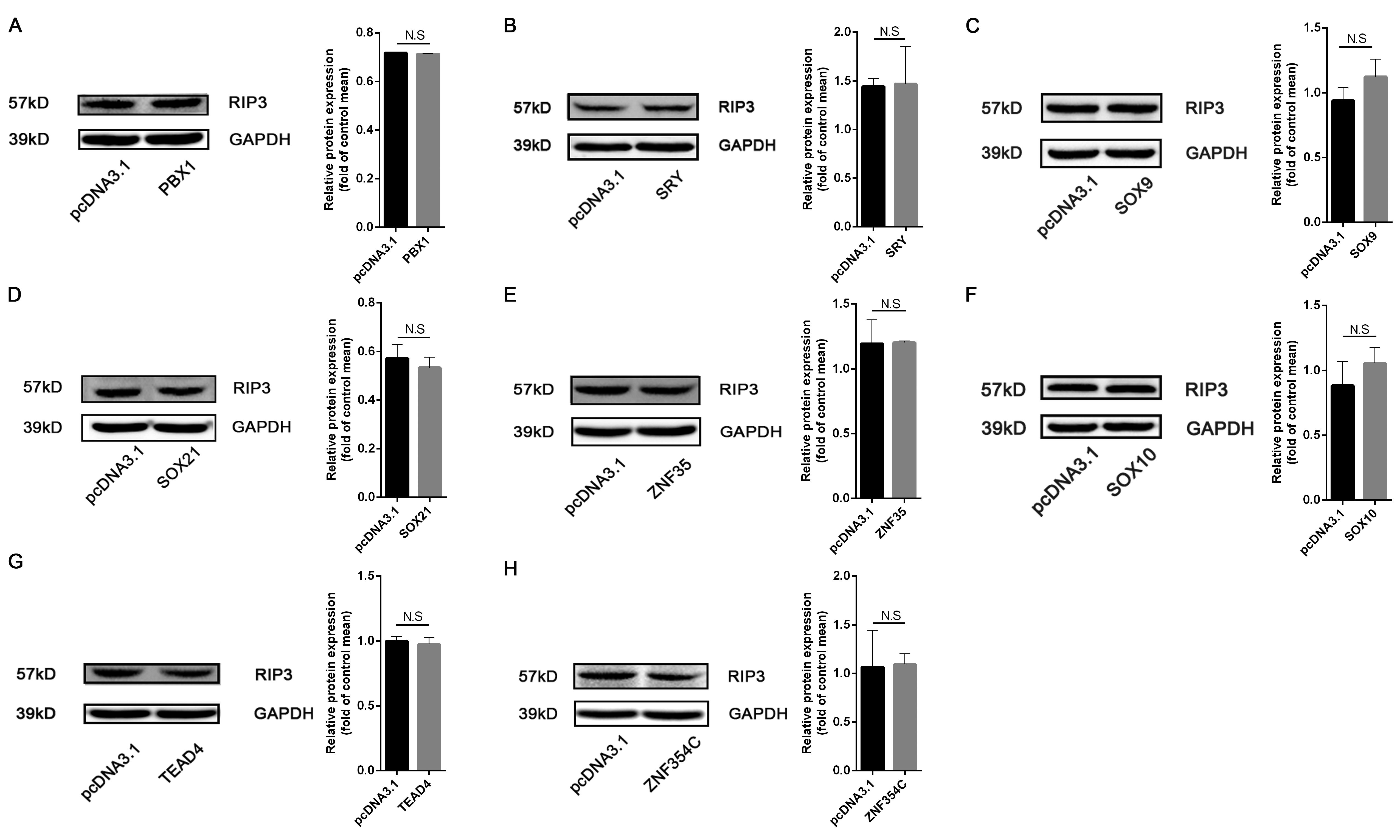

Supplement: Supplementary file 4 [file JCMM-23-5317-s004.tif]
