# Supplementary material for: A common variant of RIP3 promoter region is associated with poor prognosis in heart failure patients by influencing SOX17 binding
Source: J Cell Mol Med. 2019 May 31;23(8):5317–28. doi: 10.1111/jcmm.14408 (PMC6652837; doi:10.1111/jcmm.14408)
Supplement: Supplementary file 6 [file JCMM-23-5317-s006.docx]

| **Supplement Table 1. Sequences of PCR primers used for amplification and sequencing of human RIP3 Promoter and 5′UTR** | | | | |
| --- | --- | --- | --- | --- |
| **Region** | **Forward primers(5′→3′)** | **Reverse primers(5′→3′)** | **Tm(℃)** | **Size(bp)** |
| Promoter+5′UTR | GAAATACCCAAGTAGGCTCCAAAAG | ACGTTCTCTGAGCGAGTCTGT | 60.47 | 2251 |
| Promoter | CAGGAACCCAATCACCTCTTTC | **－** | 60 | — |
| Promoter | GCATGTGCTTGTCAGGAACATT | **－** | 60 | — |
|  |  |  |  |  |

| **Supplement Table 2. Sequence of probes and primers sets.** | | | |
| --- | --- | --- | --- |
| Rs ID | primer(5'→3') | probe(5'→3') | allele |
| rs3212250 | F 5'-GGACTGTAGAGGCGCCTATAAG-3' | 5' VIC-CGGAAAAAGGGTAACAA-MGB 3' | A |
|  | R 5'-AGGGGTCAGTCTCTAGACCAAG-3' | 5' FAM-AAAAgGGGTAgCAACCC-MGB 3' | G |
| rs3212254 | F 5'-TTGGCACCTTCGGGCAAG-3' | 5' VIC-CACCCCCCACCAGTA-MGB 3' | G |
|  | R 5'-CTGCTCCAGGCTTCAGGATC-3' | 5' FAM-CACCCCCAACCAGTA-MGB 3' | T |
| FAM, 6-carboxyfluorescein; HEX, hexachloro-6-carboxyfluorescein. MGB indicates MGB probe. | | | |

| **Supplement Table 3. Baseline Characteristics of the ELISA study Sample** | | | | | |
| --- | --- | --- | --- | --- | --- |
| **Characteristcs** | **control (n=78)** | **HF group** | | | |
|  |  | **NYHA Class II (n=70)** | **NYHA Class III (n=67)** | | **NYHA Class IV (n=70)** |
| Men, % | 50 | 51 | 50 | | 49 |
| Age, y | 63.5 | 59.2 | 55.8 | | 66.1 |
| Glucose, mmol/L | 5.16±0.43 | 6.55±1.56 | 6.07±2.28 | | 7.51±3.28 |
| TG, mmol/L | 1.30±0.64 | 1.68±1.70 | 1.23±0.68 | | 1.13±0.51 |
| TC, mmol/L | 4.72±0.99 | 3.86±1.03 | 3.78±0.87 | | 3.86±0.94 |
| HDL, mmol/L | 1.41±0.32 | 1.05±0.21 | 1.09±0.84 | | 0.91±0.28 |
| LDL, mmol/L | 2.69±0.92 | 2.29±0.79 | 2.29±0.76 | | 2.47±0.64 |
| Hypertension,% | 0 | 92 | 91 | | 96 |
| Diabetes, % | 0 | 32 | 15 | | 51 |
| Hyperlipidemia,% | 0 | 14 | 14 | | 12 |
| Smoking status, % | 0 | 27 | 29 | | 32 |
| β-blocker use，% | 0 | 69 | 38 | | 28 |
| Data are expressed as means ± SD or percentages. |  |  |  |  | |
| TG=triglyceride, TC=total cholesterol, HDL-C=high-density lipoprotein cholesterol, LDL-C=low-density lipoprotein cholesterol. | | | | | |

| **Supplement Table 4. Haploblock Structure within** RIP3 and RegulomeDB Scores for Predicted function. | | | | |
| --- | --- | --- | --- | --- |
| **Haploblock Structure** | **SNP** | **Chromosome** | **Position (hg19)** | **Regulome DB Score** |
| Haploblock 1 | rs3212251 | 14 | 24809185 | 4 |
|  | rs3212250 | 14 | 24809191 | 4 |
|  | rs3212249 | 14 | 24809415 | 2b |
|  | rs3834521 | 14 | 24809650 | 5 |
|  | rs3759625 | 14 | 24809683 | 5 |
|  | rs3212247 | 14 | 24809795 | 5 |
|  | rs3212246 | 14 | 24809850 | 5 |
|  | rs3759630 | 14 | 24810413 | 5 |
|  |  |  |  |  |
| Haploblock 2 | rs3212243 | 14 | 24810370 | 6 |
|  | rs3212242 | 14 | 24810401 | 5 |
|  |  |  |  |  |
| Haploblock 3 | rs3212245 | 14 | 24809957 | ... |
|  | rs3212241 | 14 | 24810438 | 5 |
|  | ***rs3212254**** | *14* | *24805463* | - |
|  |  |  |  |  |
| Haploblock 4 | rs1007381 | 14 | 24809611 | 5 |
|  | rs3212240 | 14 | 24810898 | ... |
| **RegulomeDB scores for predicted function (1=likely to be functional; 6= not likely to be functional). SNPs in each haploblock are in strong linkage disequilibrium (r**^2^**≥0.9** **).** rs3212254*** is the only common variant that located in the coding region of RIP3. SNPs= single nucleotide polymorphisms.** | | | | |

| **Supplement Table 5. Oligonucleotide sequences of human RIP3 promoter and transcriptional factor binding site probes for gel shift assay.** | | | |
| --- | --- | --- | --- |
|  | **Sequences (5’→3’)** | | |
| rs3212247-T-F | TTCACTACATTGTTGGATGCT | | |
| rs3212247-T-R | AGCATCCAACAATGTAGTGAA | | |
| rs3212247-C-F | TTCACTACATCGTTGGATGCT | | |
| rs3212247-C-R | AGCATCCAACGATGTAGTGAA | | |
|  |  |  |  |
